# Utilization and factors associated with antenatal, delivery and postnatal Care Services in Tigray Region, Ethiopia: a community-based cross-sectional study

**DOI:** 10.1186/s12884-020-03031-6

**Published:** 2020-06-01

**Authors:** Mussie Alemayehu, Tesfay Gebregzabher Gebrehiwot, Araya Abrha Medhanyie, Alem Desta, Tesfu Alemu, Atakelti Abrha, Hagos Godefy

**Affiliations:** 1 School of Public Health, Mekelle University, College of Health Sciences, Mekelle, Ethiopia; 2 UNFPA-Ethiopia, Tigray Branch Office, Tigray, Ethiopia; 3 Tigray Regional Health Bureaus, Mekelle, Ethiopia

**Keywords:** Antenatal care, Delivery, Institutional delivery, Postnatal care, Ethiopia, Maternal health services, Developing countries

## Abstract

**Background:**

This study aimed to identify the utilization and factors associated with antenatal care, delivery, and postnatal care services in Tigray regional state, Ethiopia.

**Methods:**

A community-based cross-sectional study was conducted among 667 women of reproductive age group who had children aged 45 days - 6 months in 13 districts (3 urban and 10 rural). Data were collected from May–June 2015. Multistage sampling technique was used. The data were analyzed using SPSS version 20. Multiple variable logistic regression analysis was used to identify the factors associated with the utilization of antenatal care, institutional delivery, and postnatal care services.

**Results:**

Of the total, the proportion of women who visited a health facility for antenatal care four or more times (ANC 4^+^) was 58.2%, those who chose institutional delivery was 87.9%, and those who received postnatal care (PNC) within 42 days of birth at least once was 40.3%. Residing in an urban area, having an electronic media, and having 2–5 children were factors associated with an ANC 4^+^ visit. Whereas, partner involvement in ANC visit (AOR = 2.4, 95% of CI: 1.37, 4.35) and content of ANC discussed (AOR = 4.0, 95% of CI: 1.08, 14.93), having birth preparedness (AOR = 2.6, 95% of CI: 1.44, 4.97), residing within a distance of less than a 30-min walk to the nearest health facility (AOR = 2.0, 95% of CI: 1.16, 3.64), and having ANC 4^+^ visits (AOR = 2.4, 95% of CI: 1.39, 4.31) were the factors that were found to be associated with institutional delivery. As regards to PNC visits within 42 days of birth, age of 40–45 years, having 2–5 children, and ANC 4^+^ visits were found to be significant factors associated with it.

**Conclusion:**

The proportion of women who attended antenatal care and gave birth in a health facility was high. However, the proportion of women who attended antenatal and postnatal care was low. Residing in urban areas, having an electronic media, living near a health facility, having partner involvement in decision making, receiving appropriate ANC counseling, having birth preparedness, age of the woman, and number of children could potentially influence maternal health services utilization.

## Background

Maternal health is a key challenge globally. The unacceptably high levels of maternal mortality are frequently discussed in global health and development meetings. Although remarkable achievement has been made, still there is little or no progress in reducing maternal death in Sub-Saharan Africa (SSA) with only a 40% reduction since 2000. SSA and Southern Asia accounted for approximately 86% (254000) of the global maternal death. In 2017, fifteen countries including Ethiopia were categorized as “very high alert” or “high alert” to maternal mortality with the peak value of 1150 per 100,000 live birth in South Sudan [1]. Ethiopia is one of the countries with high MMR estimated at 412 deaths per 100,000 live births [2]. Even though there is no single, simple or straightforward intervention that can significantly decrease maternal mortality, studies have recommended strengthening the health system, use of skilled delivery attendants, and postnatal care services [3–6].

Several factors that are related to social-cultural behavior, economic circumstances, and health systems have contributed to the overall high MMR in Ethiopia [7, 8].On the other hand, clinically, maternal mortality has been associated with unsafe abortion, hemorrhage, sepsis, or infection, a hypertensive disorder of pregnancy, and obstructed labor [4, 9]. Deaths because of these major causes could be associated with one of or a combination of the three delays: delay of deciding to seek medical care; delay of reaching health facilities after a decision is made to seek care; and delay of receiving appropriate health care services after reaching a health facility [1, 10].

The World Health Organization (WHO) has identified maternal health services such as antenatal care (ANC), institutional delivery, and postnatal care (PNC) as essential components of intervention needed to reduce maternal and child mortality [1, 3–5]. Recently, WHO recommends pregnant women should have at least 8 visits during her pregnancy [11]. Therefore, the provision of a quality continuum of maternal health services (quality care during pregnancy, access to skilled delivery and emergency obstetric care, and early postnatal care) is pivotal for the reduction of maternal deaths.

The Ethiopian Health Sector Development Program (HSDP) that has been implemented in the last 20 years gives attention to attain universal access to Primary Health Care (PHC). This includes maternal health services for the whole population through the initiatives of the Health Extension Program (HEP). HEP’s main objective is to ensure access to promotive and preventive health services. House-to-house visits are conducted by health extension workers (HEWs) to trace pregnant women for ANC follow up and to advise them to visit a health facility for institutional delivery [12–14]. To complement and support the services of HEWs, the government has introduced the women’s development army (WDA). It is a structure at the community level, comprised of one to five networks of women. WDA works together with HEW and provides services in other sectors like agriculture and education. Thus, one to five networks usually six networks create a large network of 30 women call women development groups, which has a leader and a secretary. Members of this group meet once a month to discuss health, agriculture, saving schemes, and education activities [14–16].

Various studies from the setting have reported low utilization of institutional delivery and moderate use of the ANC services at 5–6% and 54–84.6% respectively [15, 16]. However, reports published by a governmental agency (Tigray Regional Health Bureau) and peer-reviewed articles have been inconsistent. The reported inconsistencies could be due to time delays in conducting operational research. Maternal utilization of ANC and institutional delivery services might have also changed due to recent interventions. The interventions included mobilization of the community by HEWs and WDA in terms of (a) tracing all pregnant women in their localities, (b) facilitating how expectant women can reach the main road, (c) reinforcement of ambulance service in transporting expectant women from the main road to the health centers and hospitals, (d) harmonization of labor and maternal death surveillance report (MDSR) [17].

Thus, this study aims to assess the utilization and factors associated with ANC, institutional delivery, and PNC services among women of reproductive age. And it would have an input in archiving the Sustainable Development Goals (SDGs).

## Methods

### Study design and settings

A community-based cross-sectional study was used to assess utilization and factor associated with ANC, institutional delivery, and PNC services. The data was collected from May–June 2015.

The region has seven administrative zones including one especial zone, Mekelle. According to the Tigray Regional Health Bureau report, the region has 52 districts/woredas (34 rural and 18 urban) and 792 kebeles/tabias (722 rural and 70 urban). Based on the projection made from the Ethiopian census of 2007 for the year 2015, the region had a total population of 5,055,999 of whom 50.8% were females. The region is predominantly Tigrawot in ethnicity (96.5%) and Orthodox Christians in religion (95.6%) [16]. In 2014, there was one specialized hospital, 15 general hospitals, 7 primary hospitals, 224 health centers, and 668 health posts. As regards to the number and composition of health professionals, there were 107 physicians, 3861 nurses, 778 midwives, 534 health officers, and 1350 health extension workers [18]. The study was conducted among women of reproductive age (15–49 years old) who gave birth in the last 7 months in the Tigray Regional State.

### Sample size and sampling procedure

Sample size was determined for maternal health service utilization using double population proportion formula. The formula for sample size; assuming 12% postnatal coverage in Ethiopia- (19)-, 95% confidence interval, and 4% degree of precision was used. Since a multistage sampling technique was employed, a design effect of 2 was used. With the assumption of a 10% non-response rate, the total sample size computed was 696.

The surveyed districts were first listed by alphabetical order. Then 25% of the districts (13 districts/woredas) were included using a systematic random sampling technique. Rural and urban districts were represented proportionally in the ratio of 34:18 (10 rural and 3 urban). Since we have a high number of rural areas than urban, a large number of rural areas were included in the study to get more representative sampling (a large number of participants). Hence, *Tsegede, Medebay Zana, Adwa, Mereb Leke, Werei Leke, Gulo Mekeda, Degua Tembien, Emba Alaje, Raya Azebo,* and *Kilteawelalo* districts were included from a rural area. While *Abi-Adi, Endaselalsie,* and *Hawelti* districts were selected from urban areas. Besides, we systematically randomly sampled five kebele from each of the 13 districts. Thus, when the total number of sample size was divided by the number of kebeles (696/65), it resulted in 11 households from each of the selected kebeles. A spinning technique was used to identify the center of a kebele, selecting and interviewing only one woman from each of the 11 households. Women of reproductive age (15–49 years) who gave birth in the last 45 days to 6 months before the data collection were the subjects of this study. If more than one woman was present in a household, one woman was selected using a lottery method.

### Data collection and quality control

The questionnaire was adapted from the reliable, validated, and standard EDHS 2014 questionnaire [18]. It contains information on social-demographic and economic characteristics, reproductive history, and maternal health service utilization. Initially, the questionnaire was prepared in English. The English version of the questionnaire was translated to Tigrigna and back-translated to English to ensure consistency. To check the logical sequence, skip pattern, the time needed to complete the interview, acceptability of the tool, and ensure a common understanding of the tool, a pretest was done on 5% (35) women outside the study areas. It was pre-tested 2 weeks before the actual data collection. The data were collected through face-to-face interviews at comfortable and convenient places by well-trained health professionals.

### Data quality control

The investigators of this study trained and supervised the data collectors. Training and supervision of data collectors were made by the investigators. A guide was developed for data collection, implementation, and management of the survey. The investigators presented a detailed explanation of the purpose and importance of the study assuring the participants’ answers would remain confidential and to encourage them to provide honest answers. Completed questionnaires were reviewed daily for accuracy and consistency.

### Measurement

The utilization of maternal health services at health facilities is thus defined in this study when a woman visited and received care from a hospital, health center, or health post. These are ANC visits during pregnancy, institutional delivery, and PNC visit within 42 days. Number of ANC visits during pregnancy: This variable aimed to assess how many ANC visits the women had in their recent pregnancy regardless whosoever the provider of ANC service was. It was coded as ‘None’, ‘1–3’, and ‘4 and more’. Postnatal care is the care given to the mother and newborn following birth until 42 days. Its response was categorized as “yes” or “no” [18]. Institutional delivery for last-child was gathered by asking the women to the place where she gave her last birth and the response was categorized as home or institutional delivery. The response was collected into home, different governmental health facilities, different types of private health facilities, and non-governmental health facilities. Irrespective of the ownership, however, these places of deliveries were grouped into two- health institutions (governmental health facilities, private health facilities, and non-governmental health facilities and home deliveries.

ANC partner support was measured by a total of 11 items of partner support received during an ANC visit. Women who received no items of partner support were categorized as “Low ANC partner support,” women who received 1–4 items of partner support were categorized as “Moderate ANC partner support,” and women who received 5 or more items were categorized as “High ANC partner support.” ANC components discussed at a health facility was measured by a total of 18 items. Women discussing 3 or fewer items during their health care facility visit were considered low ANC component discussion, 4–7 items were moderate ANC component discussion, and 8 and above were high ANC component discussion.

Possessing electronic media was assessed among women owning mobile phones, radio, or television. Women who had at least one electronic media were labeled as “having electronic media” *whereas the remaining was labeled as “No having electronic media*”.

A family was considered a model if the household performance was certified in health (attending ANC, PNC visits, having institutional delivery, immunization, personal and environmental hygiene, nutrition, latrine construction, use of solid and liquid waste disposal mechanisms), agriculture (using fertilizer, introducing home garden, use of modern technology and getting cash crop through selling animal products), education (sending their children to formal education and participating in informal education), and microfinance (saving and credit schemes) sectors. On the other hand, for a woman who did not certify in the activities of agriculture, health, education, and microfinance, the household was considered a non-model family. Health facilities in this survey included a health post, health center, and hospital. Health posts are operational units for health extension workers and serve a population of about 5000 people. A health center is staffed with mid-level health professionals such as nurses and midwives and serves a population of about 25,000 people. One health center supervises, supports, and encompasses on average 5 health posts under its catchment area.

The residence has explained the characteristics of the women directly. Urban and rural were the responses for this variable.

### Statistical analysis

Data were entered, cleaned, and analyzed using SPSS 20 for Windows (SPSS Inc. Version 20, Chicago, Illinois). Data cleaning was done by running frequencies, cross-tabulation, and sorting among reported cases or variables. Descriptive statistics were used to summarize the data and the results were presented using frequency, tables, and percentages. A “binary analysis” was used to describe the association between independent and dependent variables and a multiple logistic regression analysis was used to show factors determining outcome variables. Before proceeding to the multiple logistic regression, variables that had a *p*-value of 0.25 or less in the binary logistic regression were included in the multivariable logistic regression. Finally, *P*-value < 0.05 was considered statistically significant for all independent variables in the *multiple variable* logistic regression.

Crude odds ratio (COR) and adjusted odds ratio (AOR) were calculated. To determine the factors most statistically significantly with maternal health services utilization, odds ratio at 95% CI was determined using logistic regression analysis. The final model was fitted using the Hosmer–Lemeshow Goodness of Fit Test. The goodness of fit of the final model was checked by using the Hosmer–Lemeshow Goodness of Fit Test and *p*-value greater than 0.05 considered as the model fit to the logistic regression.

## Result

### Socio-demographic characteristics

The response rate was 98.5%. Of the total 667 respondents, 189 (28.3%) women were between the age group of 25–29 years. A considerable number, 494 (74.1%) of the women lived within 30 min walking distance to the nearby health facilities. The mean age of the study participants was 27.5(+ 5.8) years. Three-quarters of the total, 503 (75.4%) women were from rural and around one quarter, 164(24.6%) were urban residents. Majority, 631(94.6%) were married, of which 555(83.2%) were housewives. Two hundred seventy (41.7%) of the women and 238 (35.7%) of the husbands had no formal education. Having electronic media was observed in 324 (48.6%) of the respondents. A model family was labeled in 318 (47.7%) of the households. Finally, about two-thirds of 417 (62.5%) of the study participants reported having between 2 to5 living children [Table 1].

**Table 1:** Socio demographic characteristics of women in Tigray region, 2015 (*N* = 667)

Variables (***n*** = 677)	N (%)
**Zone**	
Western	60(9.0)
North West	111(16.6)
Central	187(28.0)
Eastern	103(15.4)
South East	52(7.8)
Mekelle	57(8.5)
Southern	97(14.5)
**Distance to health facility**	
Up to 30 min	494(74.1)
Greater than 30 min	173(25.9)
**Age category of women**	
15–19	45(6.7)
20–24	175(26.2)
25–29	189(28.2)
30–34	152(22.8)
35–39	82(12.3)
40–45	25(3.7)
Mean age (SD)	27.5(±5.8)
**Marital status**	
Married	631(94.6)
Others (single, divorced and widowed)	36(5.4)
**Women’s occupation**	
Housewife	555(83.2)
Others (employed, student, and merchant)	112(16.7)
**Women education**	
No formal education	278(41.7)
Primary education	229(34.3)
Secondary education	119(17.8)
More than secondary	41(6.1)
**Husband education**	
No formal education	238(35.7)
Primary education	224(33.6)
Secondary education	135(20.2)
More than secondary	70(10.5)
**Presence of electronic media**	
Presence of radio or TV or mobile phone	324(48.6)
No electronic media	343(51.4)
**Model family**	318(47.7)
**Number of living children**	
One	165(24.7)
2–5	417(62.5)
6 and more	85(12.7)

### ANC visit

In this study, 646 (96.9%) women had at least one ANC visit at a health facility. Of these, 238(35.7%) of the women had their first visit before 16 weeks gestation of pregnancy. More than half 388(58.2%) of the total number of women had four and more ANC visits at a health facility. Of all the ANC attendants, almost four of five women 527(79%) had visited the health center for the ANC. Only 91(13.6%) women visited health posts for ANC checkups. The number of women attended by HEWs for ANC service was 48(7.2%). Measurement of weight 623(96.4%), and Blood Pressure 634(98.1%) and urine examination 611(94.6%) and folate supplementation 506 (75.9%) were the most common services provided by health professionals during ANC visit. Regarding the feeding practice, 341(51.1%) women replied that their food consumption was as usual as it had been before their pregnancy. Furthermore, three fourth (506/75.9%) of the respondents reported that they were taking iron and folic acid supplementation during their pregnancy, out of this 295(64.7%) took for less than 2 months duration [Table 2].

**Table 2:** ANC visit and type of services given at health facility with frequency and proportion of women, Tigray region, 2015

Place of ANC visit (***n*** = 667)	Number (%)
Governmental hospital	80(12.0)
Health center	527(79.0)
Health post	91(13.6)
NGO health facility	14(2.1)
Private health facility	15(2.2)
**ANC visit by type of health professional (n = 667)**	
Unspecified health professional	486(72.9)
midwives	134(21.1)
nurses	80(12.0)
HEW	48(7.2)
Others (Health officer and Medical doctor)	8(1.1)
**Number of ANC visit**	
No ANC care	21(3.1)
1	16(2.4)
2–3	233(34.9)
4 and more	388(58.2)
Do not know	9(1.3)
**Gestational age at first ANC visit**	
No ANC care	21(3.1)
Less than 4 months	238(35.7)
4–5 months	231(40.6)
6–7 months	120(18.0)
8 and more	7(1.0)
Do not know	10(1.5)
**Examination and measurement taken during ANC visit (*n*** **= 646)**	
Weight measured	623(96.4)
Height measured	281(43.5)
BP measured	634(98.1)
Urine examination	611(94.6)
Blood test	609(94.3)
HIV test	583(90.2)
STI screening	228(35.3)
Taking Iron tablet	553(85.6)
General examination	543(84.3)
MUAC	250(40.1)
**Amount of food taken during pregnancy (667)**	
Less than usual	222(33.3)
As usual	341(51.1)
More than usual	101(15.1)
Unable to decide	3(0.4)
**Duration of Iron supplementation during pregnancy (months) (*n*** **= 456)**	
Less 2 months	295(64.7)
Three months	124(27.2)
Four months and more	37(8.1)

**Fig. 1:**
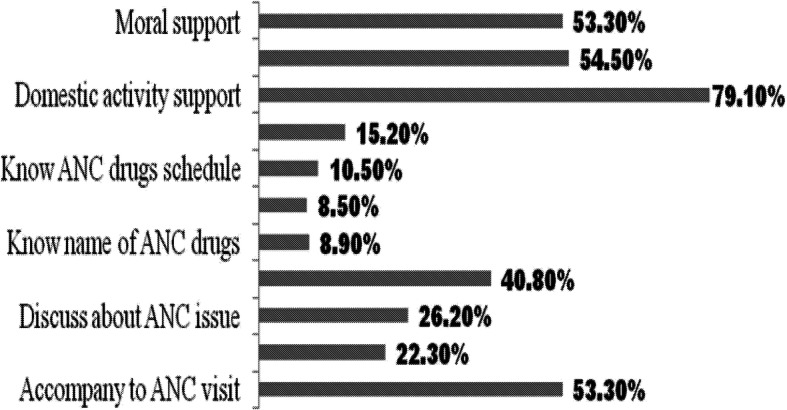
Type of ANC partner support in Tigray region, 2015

Seventy-six percent of the women received at least one kind of partner support during their pregnancy. The most common partner’s support was related to accompanying a woman during ANC visit 263(53.3%), performing/doing domestic activities 390(79%), and having joint decision 268(54.5%) [Fig. 1]. Furthermore, the composite measure of ANC partner support indicated that 171(25.6%), 392(58.5%) and 104 (15.6%) of women got low, moderate and high ANC partner support, respectively during their visit.

### Birth preparedness

More than eight of 10 women, 583(87.4%) of the women practiced the birth preparedness plan of their last pregnancy. The three common types of activities mentioned were preparing food, 562(96.4%), saving money 342(58.7%), and arranging transport 248(42.5%). Majority of women, 567 (97.3%) preferred to be attended by nurses, midwives, and doctors for their last pregnancy. Most women in study 414 (74.1%) planned for delivery at the health center. Furthermore, the majority of 510 (90.6%) of the women made their own decision for the place of birth [Table 3].

**Table 3:** Birth preparedness of women during their last pregnancy in Tigray region, Ethiopia, 2015

Preparedness made for last pregnancy (***n*** = 583)	Number (%)
Money	342(58.7)
Transport	248(42.5)
Food	562(96.4)
Identified companion	105(18.0)
Identified place of birth	242(41.5)
Prepared clean materials for delivery	243(41.7)
Identified someone for blood donation	42(7.2)
**Plan for birth assistant (n = 583)**	
Health professional (nurse, midwives and doctors)	567(97.3)
HEW	16(2.7)
Others (TTBA, mother, mother-in-law and HDA)	14(2.3)
**Plan for place of birth(*n*** **= 563)**	
home	8(1.4)
Government hospital	120(21.3)
Health center	417(74.1)
Health post	14(2.5)
Others (NGO health facility and Private hospital)	4(0.8)
**Decision on place of birth (n = 563)**	
Self	510(90.6)
husband	96(17.1)
mother	24(4.3)
Mother-in-law	20(3.6)
grandmother	6(1.1)
HEW	77(13.7)
Women development army	16(2.8)
Health professionals	87(15.5)

### Delivery service

**Fig. 2:**
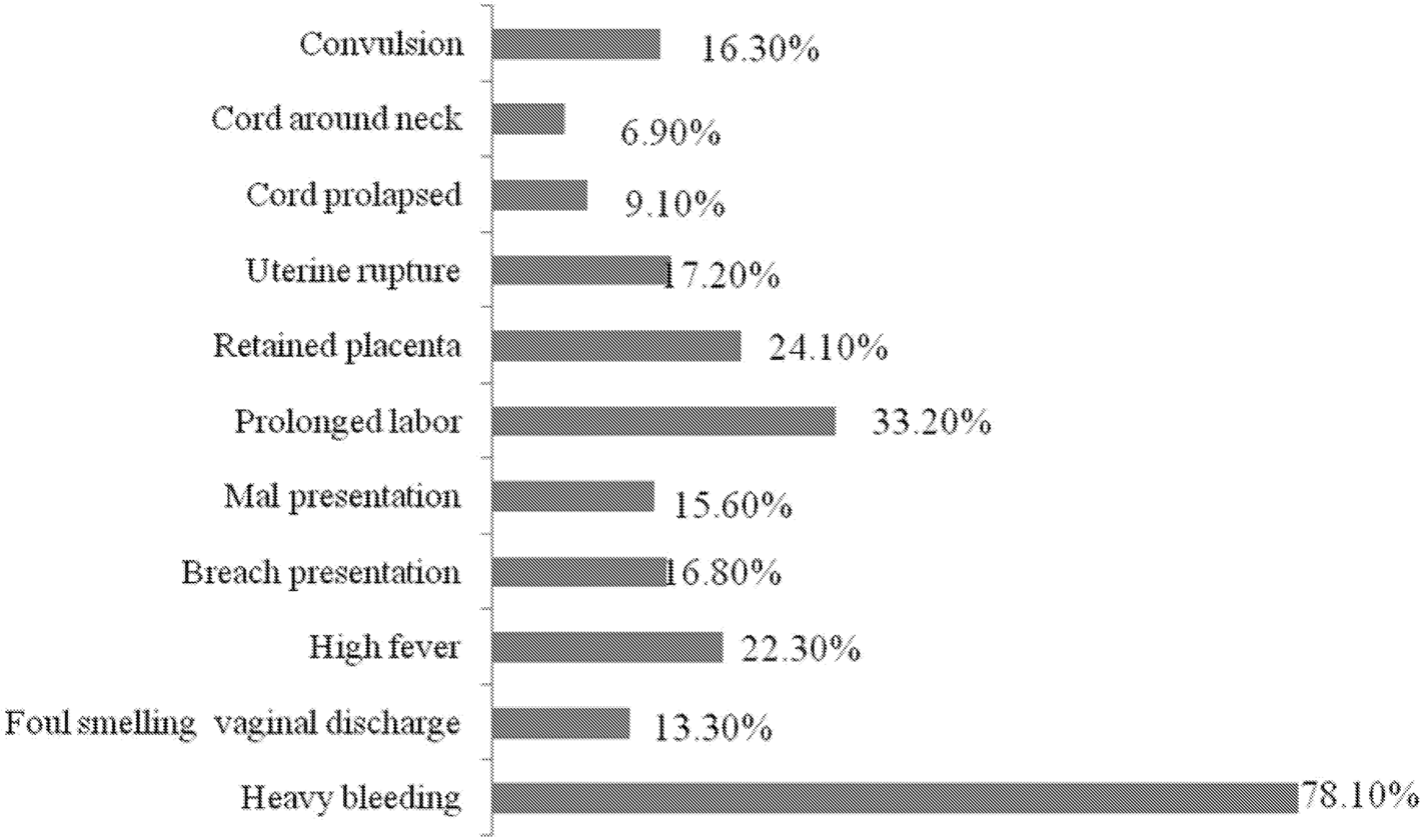
Women’s knowledge of danger signs of pregnancy and childbirth in Tigray region, 2015

A total of 586 (87.9%) women gave birth in a health facility. Of those women who gave birth in a health facility, 571 (97.4%) were assisted by skilled birth attendants, of which 15(2.6%) was attended by HEWs. Three fourths, 494(74.1%) of the respondents received partner support during delivery. The commonest type of supports mentioned by the participants was financial 385(77.9%) and accompanying during delivery, 351(71.1%). Five hundred sixty-one (84.1%) of the women were aware that they should seek treatment for danger signs of pregnancy. Of the total 34 women who developed a complication during their last birth, one-in-two (17/50.0%) had bleeding. More than half of the respondents who developed a complication sought treatment from the hospital (Table 4). Women’s knowledge of danger signs of pregnancy and childbirth indicates that bleeding was mentioned by three-fourths of the participants. The other three common types of danger signs reported by participants were prolonged labor (33.2%), retained placenta (24.1%), and having a high fever (22.3%) [Fig. 2]. Adequate information about essential newborn care practice was reported by the majority of the women; 654(98.1%) breastfed exclusively for the first 3 days, 569(85.3%) gave colostrum to the baby and 530 (79.5%) initiated early breastfeed for their newborn.

**Table 4:** Delivery service utilization and knowledge of women on danger signs and during childbirth, Tigray region, 2015

Place of delivery (***n*** = 667)	Number (%)
Home	81(12.1)
Governmental hospital	155(23.2)
Health center	411(61.6)
Health post	15(2.2)
Others (NGO and private health facilities)	5(0.7)
**Partner support**	494(74.1)
Type of support(*n* = 494)	
Arrange ambulance	307(62.1)
Accompany during delivery	351(71.1)
Stayed with me during delivery	307(62.1)
Financial support	385(77.9)
Moral support	335(67.8)
**Knowledge on place for seeking treatment on danger sign**	
Governmental hospital	291(43.6)
Health center	561(84.1)
Health post	60(9.0)
NGO health facilities	22(3.3)
private health facilities	49(7.3)
Women who faced complication during last birth	34(5.1)
**Complication type (*n*** **= 34)**	
Heavy bleeding	17(50.0)
Foul smelling vaginal discharge	2(5.9)
High fever	5(14.7)
Breach presentation	3(8.8)
Mal presentation	7(20.6)
Prolonged labor	9(26.5)
Retained placenta	3(8.8)
Uterine rupture	1(2.9)
Cord prolapsed	1(2.9)
convulsion	1(2.9)
**Place sought for treatment of danger sign(n = 34)**	
Governmental hospital	20(58.8)
Health center	12(35.3)
Health post	1(2.9)
private health facilities	1(2.9)

### Postnatal care (PNC)

Only 222 (33.3%) of women were visited at their homes for postnatal services by health care workers. Of the participants who received PNC services at home, 194 (87.4%) were visited by HEWs, and 30 (13.5%) by nurses. However, a small proportion of 14 (2.1%) of women visited health facilities within 2 days for PNC. Majority of women 307 (76.9%) attended PNC at the health center. Regarding the frequency of visits, 269 (40.3%) women had visited health facilities once and 60 (9.0%) women had visited twice within 42 days [Table 5].

**Table 5:** Women’s postnatal care utilization, type of health workers and time of visit, Tigray region, 2015

Variables	Home	Health facility
**PNC visit by type of health workers**	Number (%)	Number (%)
HEW	194(87.4)	21(5.3)
Nurse	30(13.5)	80(20.1)
Midwives	18(8.1)	44(11.0)
Health officer	–	2(5.0)
I do not know	–	285(71.4)
**Time of first PNC visit (n = 667)**		
No visit	445(66.7)	269(40.3)
Within two days	31(4.6)	14(2.1)
3–6 days	87(13.0)	31(4.6)
7–41 days	95(14.2)	224(33.6)
42 and above days	9(1.3)	129(19.3)
**Time of second PNC visit at home (n = 667)**		
No visit	561(84.1)	431(64.6)
Within two days	4(0.6)	0(0.0)
3–6 days	13(1.9)	11(1.6)
7–41 days	76(11.4)	49(7.3)
42 and above days	13(1.9)	176(26.4)
PNC at least one visit within 42 days(n = 667)	213(31.9)	269(40.3)
PNC second visit within 42 days(n = 667)	93(13.9)	60(9.0)

On the other hand, nearly two-thirds of the women 432 (64.7%) had ever visited health facilities for at least one postnatal care visit within 42 days.

More than three-quarters of the women, 307 (76.9%) had attended their PNC at the health center, whereas only 51 (12.8%) attended at the health post. Regarding the frequency of visits, 269 (40.3%) women visited health facilities once and 60 (9.0%) women visited twice within 42 days. Besides, 469 women (70.3%) reported their children received Vitamin A supplementation [Table 5].

### Factors associated with the utilization of ANC 4+ services

Place of residence (urban versus rural) was the most significant predictor of utilization of ANC services beyond four times. The odds of having ANC 4**+** visit was almost two times higher among women residing in urban than rural areas (AOR = 1.7, 95% of CI: 1.07, 2.87). The presence of at least one electronic media was found to be a significant predictor for the utilization of ANC services. Women who had at least one electronic media had higher odds of ANC 4+ visit utilization as compared with women who don’t have electronic media (AOR = 1.6, 95% of CI: 1.13, 2.29). Besides, the odds of ANC 4+ visit was higher among women who had two up to five children as compared with women who had six and above children (AOR = 1.7, 95% of CI: 1.07, 2.87) [Table 6].

**Table 6:** Factors associated with ANC 4+ visits by women in Tigray region, 2015

Variables	ANC 4+ visit		COR [95% CI]	AOR [95% CI]
	Yes	No		
	N (%)	N (%)		
**ANC Partner support**				
Low	90(52.6)	81(47.4)	0.7(0.46,1.23)	1.0(0.56,2.03)
Moderate	236(60.2)	156(39.8)	1.0(0.65,1.59)	1.3(0.77,2.49)
High	62(59.6)	42(40.4)	1	1
**Contents of ANC discussed**				
few	158(53.2)	139(46.8)	0.6(0.43,1.08)	0.5(0.32,1.09)
Moderate	167(62.1)	102(37.9)	0.9(0.60,1.58)	0.7(0.42,1.39)
High	63(62.4)	38(37.6)	1	1
**Number of Living children**				
One	97(58.5)	68(41.4)	1.9(1.14,3.3)	1.5(0.85,2.62)
Two up to Five	255(61.2)	162(38.8)	2.1(1.33,3.43)	**1.7(1.07,2.87)**
Six and above	36(42.4)	49(57.6)	1	1
**Residence**				
Urban	154(69.4)	68(30.6)	2.1(1.5,3.00)	**1.7(1.07,2.87)**
Rural	216(51.6)	203(48.4)	1	1
**Having at least one electronic media**				
Yes	217(67.0)	107(33.0)	2.0(1.34,2.67)	**1.6(1.13,2.29)**
No	171(49.9)	172(50.1)	1	1

### Factors associated with institutional delivery

Partner support during ANC service contributed to enhanced institutional delivery. Thus, moderate than low partner support in the utilization of ANC service greatly enhanced institutional delivery (AOR = 2.4, 95% of CI: 1.37, 4.35). Discussing the contents of ANC services between the woman and health care provider also greatly increased institutional delivery (AOR = 4.0, 95% of CI:1.08, 14.93).

Place of residence (urban Vs rural) was reported to be a significant predictor of institutional delivery. The odds of having institutional delivery in urban women were three times greater than their rural counterparts (AOR = 2.7, 95% CI: 1.23, 6.0). Besides, the presence of at least one electronic media was found to be a higher odd of institutional delivery than women who don’t have at least one electronic media (AOR = 2.2, 95% of CI: 1.18, 4.39). The odds of institutional delivery were higher among women who had birth preparedness than those who had no plans (AOR = 2.6, 95% of CI: 1.44, 4.97). Women who lived within a walking distance of less than 30 min to the nearest health facility were two times more likely to deliver at the health institution (AOR = 2.0, 95% of CI: 1.16, 3.64). Furthermore, the number of ANC visits was also found to be a predictor of institutional delivery. Mothers who had four and above visits were two times more likely to deliver at the health facility than those who had less (AOR = 2.4, 95% of CI: 1.39, 4.31) [Table 7].

**Table 7:** Factors associated with institutional delivery of women in Tigray region, 2015

Variables	Place of Delivery		COR [95%]	AOR [95%]
	Institution	Home		
	N (%)	N (%)		
**Partner support**				
Low	133(77.8)	38(22.2)	1	1
Moderate	355(90.6)	37(9.4)	2.7(1.67,4.49)	**2.4(1.37,4.35)**
High	98(42.6)	6(5.8)	4.6(1.89,11.47)	1.5(0.50,5.05)
**Contents of ANC discussed**				
Low	246(82.8)	51(17.2)	1	1
Moderate	243(90.3)	26(9.7)	1.9(1.17,3.2)	1.5(0.84,2.74)
High	97(96)	4(4.0)	5.0(1.76,14.28)	**4.0(1.08,14.93)**
**Number of Living children**				
One	154(93.3)	11(6.7)	3.7(1.68,8.39)	**3.2(1.30,8.10)**
2–5	365(87.5)	52(12.5)	1.8(1.03,3.42)	1.3(0.67,2.60)
Six and above	67(78.8)	18(21.2)	1	1
**Residence**				
Urban	213(95.9)	9(4.1)	4.5(2.1,5.7)	**2.7(1.23,6.0)**
Rural	351(95.8)	68(16.2)	1	1
**Having at least one electronic media**				
Yes	309(95.4)	15(4.6)	4.9(2.73,8.79)	**2.2(1.18,4.39)**
No	277(80.8)	66(19.2)	1	1
**Birth Preparedness**				
Yes	524(90.8)	53(9.2)	4.4(2.63,7.57)	2.6(1.44,4.97)
No	62(68.9)	28(31.1)	1	1
**Distance to health facility**				
Within 30 min	442(89.5)	52(10.5)	1.7(1.04,2.79)	2.0(1.16,3.64)
30 min and above	144(83.2)	29(16.8)	1	1
**Number of ANC visit**				
Less than 4	222(79.6)	57(20.4)	1	1
Four and above	364(93.8)	24(6.2)	3.8(2.34,6.45)	2.4(1.39,4.31)

### Factors associated with the first PNC visit within 42 days

Age was a significant predictor for using the first PNC visit within 42 days. The odds of having the first PNC visit within 42 days were almost four times higher among women aged 40–45 years than 15–19 years (AOR = 3.8, 95% CI:1.12,12.85). The odds of having the first PNC visit within 42 days was 2 times higher among women children between 2 and 5 than ≥6 children (AOR = 2.0, 95% of CI: 1.08, 3.81). Moreover, the number of ANC visits was found to be a predictor for making the first PNC visit within 42 days. Thus, the odds of having first PNC visit was higher among women who had four and above ANC visit than women who had less than four visits (AOR = 1.6, 95% of CI: 1.15, 2.26) [Table 8].

**Table 8:** Factors associated with First PNC within 42 days of women in Tigray region, 2015

Variables	PNC 1st visit Within 42 days		COR [95%]	AOR [95%]
	Yes	No		
	N (%)	N (%)		
**Number of Living children**				
One	61(37.0)	104(63.0)	1.3(0.76,2.32)	1.6(0.77,3.66)
Two up to five	182(43.6)	235(56.4)	1.7(1.06,2.89)	**2.0(1.08,3.81)**
Six and above	26(30.6)	59(69.4)	1	1
**Age**				
15–19	15(33.3)	30(66.7)	1	1
20–24	71(40.6)	104(59.4)	1.3(0.68,2.72)	1.1(0.53,2.33)
25–29	77(41.0)	111(59.0)	1.3(0.7,2.75)	1.1(0.50,2.47)
30–34	64(42.1)	88(57.9)	1.4(0.72,2.92)	1.2(0.55,2.99)
35–39	28(24.1)	54(65.9)	1.0(0.48,2.23)	1.2(0.47,3.09)
40–45	14(56.0)	11(44.0)	2.5(0.93,6.94)	**3.8(1.12,12.85)**
**Distance to health facility**				
Within 30 min	208(42.1)	286(57.9)	1.3(0.93,1.91)	1.3(0.91,1.92)
30 min and above	61(35.3)	112(64.7)	1	1
**ANC visit**				
Less than 4 visits	93(33.3)	186(66.7)	1	1
4 and above	176(45.4)	212(54.6)	1.6(1.2,2.28)	**1.6(1.15,2.26)**
**Place of delivery**				
Institution	241(41.1)	345(58.9)	1	1
Home	28(34.6)	53(65.4)	0.7(0.46,1.23)	0.8(0.53,1.48)

## Discussion

The study assessed utilization and factors associated with maternal health services (ANC, delivery service, and PNC) among women of reproductive age, who had children aged from 45 days to 6 months. The study covered a quarter of the districts (13 districts) in the region. Rural and urban districts were represented proportionally. The maternal health services utilization was 58.2% for ANC visits of 4 and more, 88% for institutional delivery, and 40.3% for PNC service within 42 days. Factor associated with ANC 4+ visit on multiple logistic regression analyses were place of residence, possession of electronic media, and number of children. Partner involvement in the decision to use of ANC services, the contents of ANC discussed during the visit, residence (urban vs rural), birth preparedness, and ANC 4+ visit were factors associated with institutional delivery. Age of the women, number of children, and number of ANC visits were factors associated with the first PNC visit within 42 days.

The utilization of the first PNC service within 2 days after delivery was only 2.1%. Although we reported remarkable progress in the utilization of ANC and institutional delivery, the PNC service remains low. This finding is consistent with the report of Mini EDHS 2014. It was reported that the utilization of the first PNC visit within 24–48 h was low in Amhara (0.5%), Somali (2.6%) and Oromia (2.6%) regions [18]. Based on the WHO report the first 24–48 h of birth is of the greatest need for both the mother and the baby. Consequently, approximately half of women and 30–50% of neonates die within this period [19, 20]. The possible explanation for the low first PNC visits within 2 days of delivery might be due to little attention by the health workers at the grass-root level after a successful birth. Health workers seem to focus more on the survival of the woman during ANC and delivery than at PNC services. Attention given and efforts made by HEWs and WDGs to improve the utilization of the ANC and delivery don’t happen after a mother gave birth. This could be one of the reasons for the very low coverage and provision of immediate PNC services.

In comparison to the results of the Ethiopian mini EDHS 2014 and other previous studies, our findings of the utilization of ANC visit of 4^+^ (58.2%), institutional delivery service (88%) and PNC service of the first visit within 42 days (40.3%) were remarkably higher [15, 16, 18, 21–23]. The better ANC and institutional delivery service utilization were also reported in a recent study [24]. This remarkable improvement in maternal health services utilization over a short period may be attributed to the high level of political commitment and sustained community mobilization through the HEP and WDA [25]. Besides, the Ethiopian Federal Ministry of Health (FMoH) strategy for the identification of any maternal death at the grassroots level within the concept of a maternal death surveillance report (MDSR) might have contributed to the increased service utilization. The government of Ethiopia has also introduced a new strategy for community mobilization known as WDA and commonly referred to as one of five networks for women to discuss and share their experiences on health-related issues [26]. These interventions may have contributed to the remarkable increment of institutional delivery from 25 to 88%. However, the sustainability of these interventions would require a continuous political commitment to maintain the achievements registered within a few years. This might be ensured by empowering women to bring a behavioral change and seeking maternal health series utilization without the influence of their husbands. Given that many studies have documented the achievements of the interventions, further studies must identify the best practices and strategies that contributed to the success of replicability in other settings.

This study reported that having one of electronic media was a significant predictor of the use of ANC 4^+^ service. The presence of the media might expose women with information on the importance of the ANC and other health-related information. This might have influenced women to develop positive behavior towards health service utilization. Therefore, it would be helpful to disseminate health information through electronic media. In particular, these days the proportion of women with mobile phones and places with mobile network coverage is increasing even in rural areas. Thus, taking advantage of such technologies may improve maternal health services utilization.

Place of residence was found to be a significant factor associated with ANC 4^+^ and institutional delivery. ANC should be received early in the pregnancy and continued until delivery to minimize or rather avoid adverse pregnancy outcomes. Referring to WHO recommendation a woman without complications should have at least four antenatal visits, the first of which should take place during the first trimester [3]. However, our findings on institutional delivery were similar to previous studies [22, 23, 27]. Easy access to most health facilities, highly educated creating better awareness, and improving the infrastructure of urban than rural residents, led to better utilization of maternal health services.

The ANC services are the most important contact point for mothers with health care providers, where information about risks related to pregnancy and delivery might be provided. Furthermore, ANC service utilization is a significant factor associated with a woman’s choice of where to deliver [3]. Hence in this study, we confirmed that a woman who had four or more visits was more likely to deliver at the health facility and attend her first PNC visit within 42 days of delivery. Our finding is in agreement with WHO guidelines, which recommends increasing the number of ANC visits since there is strong evidence with planning and having institutional delivery. This finding is also similar to other previous studies [21–23, 28].

Partner involvement in the ANC care and discussion of the contents of ANC during the visit of mother to a health facility was significantly associated with institutional delivery. Involving the husband in ANC service could be one potent factor for enhancing institutional delivery in developing countries like Ethiopia, where the husbands are mostly the breadwinners. Furthermore, the contents of services offered and the kind of information given to the woman during ANC visits are also important components of quality care. These services raise awareness of the danger signs during pregnancy, delivery, and postnatal period. Thus improving their health-seeking behavior, and reinforces them to practice birth preparedness [3]. This is reaffirmed by this study since we reported that mothers who discussed the contents of the ANC with the health care providers were more likely to deliver at the health institution. This might imply that providing a woman with comprehensive information on the contents of ANC could build their knowledge and enhances institutional delivery.

Although this study included 13 districts in the Tigray region, Ethiopia, women living in very remote areas with no access to transportation or far from an hour walking distance from the center of the selected villages (Kebeles) were not reached. Income level of women which is one of the factors that affect maternal health service utilization was not also directly assessed. Rather, we tried to assess the income level indirectly through the presence of electronic media by assuming that people with good income will have a better chance of possessing one of the electronic media. Thus, the findings of this study should be interpreted with due consideration of these limitations.

## Conclusion

The maternal health service, including 4 or more ANC visits and institutional delivery among women who gave birth within the 6 months before the study period was high. Despite the high coverage of 4 or more ANC visits and institutional delivery, the postnatal care services within 2 days and 42 days after birth was low. Residing in urban areas, possession of electronic media and the number of children could influence the utilization of four or more ANC visits, whereas having partner involvement in the decision to use ANC service, the distance of walking time of less than 30 min to the nearest health facility, discussing the contents of the ANC during a visit, residing in urban areas, having birth preparedness and four or more ANC visits were the factors making a difference in institutional delivery. All actors need to collaborate for keeping the momentum in mobilizing the HEP and women’s development groups to readdress the improved utilization of the early PNC visit.

## Data Availability

The dataset supporting the conclusions in this article is included in this article (tables and figures). The SPSS dataset is available from the principal investigator of the study (MA) and can be shared upon an official request. However, the SPSS dataset cannot be shared online or to a third party as we assured the research participants that the information provided will be kept confidential.

## Abbreviations

ANC: Antenatal Care
AOR: Adjusted Odds Ratio
EDHS: Ethiopian Demographic Health Survey
HEP: Health Extension Program
HEWs: Health Extension Workers
HSDP: Health Sector Development Program
MDSR: Maternal Death Surveillance Report
PHC: Primary Health Care
PNC: Postnatal Care
SSA: Sub-Saharan Africa
WDA/G: Women Development Army/Group
WHO: World Health Organization
